# The aminoglycoside antibiotic gentamicin is able to alter metabolic activity and morphology of MDCK-C11 cells: a cell model of intercalated cells

**DOI:** 10.1590/1414-431X20187417

**Published:** 2018-08-16

**Authors:** A.G.G. Coutinho, S.M.P. Biscaia, R. Fernandez, A.L. Tararthuch

**Affiliations:** 1 Departamento de Fisiologia, Setor de Ciências Biológicas, Universidade Federal do Paraná, Curitiba, PR, Brasil; 2 Departamento de Biologia Celular, Setor de Ciências Biológicas, Universidade Federal do Paraná, Curitiba, PR, Brasil

**Keywords:** Gentamicin, [Ca^2+^]_i_, MDCK-C11 cells, Cytotoxicity

## Abstract

It is well known that the aminoglycoside antibiotic gentamicin is capable of causing damage to kidney cells. Given the known involvement of Ca^2+^ in the nephrotoxic action of gentamicin, the purpose of this study was to establish a relationship between the concentration of intracellular Ca^2+^ ([Ca^2+^]_i_) and cellular cytotoxicity using MDCK-C11 cells, a clone that has several properties that resemble those of intercalated cells of the distal nephron. Changes in [Ca^2+^]_i_ was determined using fluorescence microscopy. Cell viability was evaluated by the neutral red method, and cell cytotoxicity by the MTT method. The [Ca^2+^]_i_ gradually increased when cells were exposed to 0.1 mM gentamicin for 10, 20, and 30 min. The presence of extracellular Ca^2+^ was found to be necessary to stimulate the increase in [Ca^2+^]_i_ induced by gentamicin, since this stimulus disappeared by using 1.8 mM EGTA (a Ca^2+^ chelator). Morphological changes were observed with scanning electron microscopy in epithelial cells exposed to the antibiotic. Furthermore, with the MTT method, a decrease in metabolic activity induced by gentamicin was observed, which indicates a cytotoxic effect. In conclusion, gentamicin was able to alter [Ca^2+^]_i_, change the morphology of MDCK-C11 cells, and promote cytotoxicity.

## Introduction

Gentamicin is an aminoglycoside antibiotic that has a broad spectrum of action against gram-negative bacterial infections and it is more recommended in severe cases (1). Since their development in 1940, aminoglycosides are preferably used in antimicrobial therapy. Gentamicin was discovered in 1963 and is still widely used, despite the availability of other aminoglycosides. Gentamicin remains popular because it is a low-cost, quite effective antibiotic, demonstrating low antimicrobial resistance (2). However, its use is restricted because of the development of ototoxicity and nephrotoxicity. Many studies were designed to reveal the underlying mechanism of its ototoxic and nephrotoxic effects. Currently, gentamicin-induced nephrotoxicity occurs in about 10 to 20% of treatments, and the progression to acute renal failure typically manifests after 5-7 days of treatment (3). In patients treated for more than 14 days, nephrotoxicity appears in about 50% of them, resulting in increased morbidity during and after treatment (4).

The proximal tubule is the first nephron segment that undergoes structural and histological changes caused by gentamicin and it is the major site of drug accumulation. Gentamicin leads to the loss of brush border integrity and evident apoptosis and necrosis of proximal tubular cells (5). Accumulation of gentamicin in proximal tubule cells is due to the existence of an apical membrane endocytic complex involving the proteins megalin and cubilin (6). This endocytic receptor complex transports cationic molecules present in the tubular ultrafiltrate into the cells. Once inside the cells, gentamicin alters the function of various organelles. In addition to its toxic effects exerted through endocytosis, gentamicin is able to activate a calcium-sensing receptor (CaSR) present in the apical membrane of proximal tubular cells, which has been associated with tubular cell death (3). CaSR agonists such as gadolinium and gentamicin activate phospholipase C in proximal tubular cells, resulting in the formation of inositol 1,4,5-trisphosphate and consequent Ca^2+^ release from intracellular stores (7). Recent studies have pointed to a central role of Ca^2+^ in the regulation of programmed cell death (3,8).

There are few findings about the effect of aminoglycosides on distal kidney cells. Some of these studies determined endocytosis-independent mechanisms by which gentamicin appears to directly cross the plasmatic membrane into the cytoplasm with the participation of membrane receptors such as the non-selective cation receptor TRPV (9,10 [Bibr B11] [Bibr B12]). It is well established that MDCK-C11 cells, a recently cloned subtype of Madin-Darby canine kidney cells akin to intercalated cells of the collecting duct, also have TRPV and CaSR receptors (9–13). However, the effect caused by gentamicin in these cells is not yet well understood. Understanding the cellular mechanisms by which the nephrotoxic aminoglycoside gentamicin activates membrane receptors and induces cellular cytotoxicity will enable future identification of aminoglycoside-sensitive targets and potential uptake blockers (14).

Given the known involvement of Ca^2+^ in the nephrotoxic action of gentamicin in proximal tubules, the purpose of this study was to establish a relationship between the concentration of intracellular Ca^2+^ ([Ca^2+^]_i_) and cellular cytotoxicity, using MDCK-C11 cells, a clone that has several properties that resemble those of intercalated cells of the distal nephron.

## Material and Methods

### Cell culture

A subtype (clone) of MDCK cells denominated C11 (MDCK-C11), obtained from Dr. H. Oberleithner (Department of Physiology, University of Münster, Germany), was cultured in MEM (Gibco, USA), supplemented with 10% fetal bovine serum, 100 IU/mL penicillin, 10 mg/mL streptomycin, and 2 g/L NaHCO_3_. Cells were grown at 37°C (pH 7.4) in a humidified 95% air and 5% CO_2_ incubator (Shel Lab, USA). These cells were used from passages 75 to 90.

### Intracellular Ca^2+^ measurements

Qualitative changes in [Ca^2+^]_i_ cell cultures were monitored for FLUO-4 fluorescent dye intensity emission. The excitation spectrum for this fluorescent marker is 480 nm, whereas the fluorescence was measured at 520 nm every 1 second. The data are reported in arbitrary units of fluorescence (AUF). The resulting changes in fluorescence intensity during the experiments are reported as percentages relative to the fluorescence intensity at baseline (recording period with control solution: 135 mM NaCl, 5 mM KCl, 1 mM NaH_2_PO_4_, 1 mM Na_2_SO_4_, 1.8 mM CaCl_2_, 10 mM HEPES, 1 mM MgCl_2_, 10 mM glucose). Cells grown to confluence on glass coverslips were incubated for 20 min with 10 µM FLUO-4 (Molecular Probes, USA). After this, the glass coverslips were rinsed and placed into a thermoregulated chamber (37°C) assembled on an inverted epifluorescense microscope (Axio Observer Z1, Carl Zeiss, Germany). Areas of interest containing MDCKC11 cells were identified at 20× magnification using the software AxioVision 4.8.1. Following a stabilization period with the control solution, the cell monolayer was exposed for 10, 20, and 30 min to a control solution containing 0.1 mM gentamicin sulfate (Santa Cruz Biotechnology, USA) with a recording period of about 1 min. Effects of the CaSR agonist 0.3 mM Gd^3+^, the calcium chelating agent 1.8 mM EGTA (ethylene glycol-bis(β-aminoethyl ether)-N,N,N′,N′-tetraacetic acid; Amresco, USA), and 50 mM BAPTA (Santa Cruz Biotechnology) were evaluated under the same conditions compared to the effects of gentamicin.

### Analysis of cell viability and cell cytotoxicity

Cell viability was evaluated by the method of neutral red, performed according to the protocols (15,16 [Bibr B17]). This method evaluates the cell ability for endocytosis by the uptake of the dye neutral red, by fluid phase. Cell cytotoxicity was evaluated by the MTT method (thiazolyl blue tetrazolium bromide, M5655, Sigma, USA), according to protocols previously described (16–18). The MDCK C11 cells were maintained in 96-well plates containing 0.1 mM gentamicin sulfate for 30 min, 1, 3, 24, and 48 h. These plates were subjected to reading absorbance (540 nm for neutral red and 550 for MTT) in a microplate reader (Biotek, EPOCH, USA.

### Morphological analysis

MDCK C11 cells were seeded onto sterile 13-mm round cover slips plated in 24-well plates. After different times of treatment, cells were washed 4 times in 0.1 M cacodylate buffer, pH 7.4 at 4°C. Next, the cells were fixed in Karnovski solution (2.0% glutaraldehyde, 4% paraformaldehyde, 1 mM CaCl_2_ in 0.1 M cacodylate buffer, pH 7.4) for 1 h and washed 3 times in cacodylate buffer. Then they were post-fixed (1% osmium tetroxide in sodium cacodylate buffer 0.1 M, pH 7.4) for 1 h at room temperature and washed again in 0.15 M cacodylate buffer, pH 7.4. The MDCK-C11 cells were then dehydrated in increasing concentrations of ethanol (Merck, Germany) (30, 50, 70, 90, and twice 100%) for a period of 10 min at each concentration. Finally, the cells were subjected to critical point in CPD 030 equipment, gold-coated in the SCD 030 equipment (Balzers, USA) and were analyzed in an LV scanning electron microscope (JEOL JSM 6360, USA) at UFPR Electron Microscopy Center.

### Statistical analysis

Statistical analysis was performed using the GraphPad InStat 3.10 (GraphPad Software, USA). Data are reported as means±SD. In all data of [Ca^2+^]_i_, measurements are reported as number of areas/number of assays. For analyzes between two groups, Student's *t*-test for paired samples was used. For analysis of more than two groups, we performed one-way ANOVA, followed by the Student-Newman-Keuls contrast test. Differences were considered significant if P<0.05.

## Results

### Effects of gentamicin on [Ca^2+^]_i_

**Figure 1.**
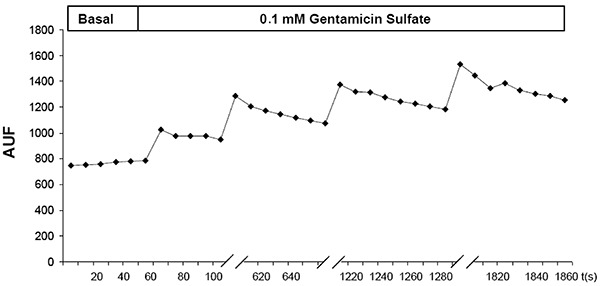
Representative recording corresponding to variations in [Ca^2+^]_i_. After a basal period, cells were exposed to 0.1 mM gentamicin sulfate. At each pause of 10 min, new excitation of the fluorophore was recorded, completing 30 min of gentamicin incubation. AUF: arbitrary units of fluorescence.

**Figure 2.**
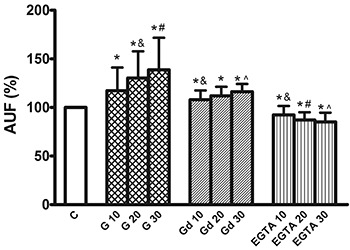
Mean values ±SD of percentage change in fluorescence (AUF), which indicates changes in [Ca^2+^]_i_ compared to a hypothetical average (C=control 100%). G 10 (n=39/7), G 20 (n=45/8), and G 30 (n=44/8) correspond to the [Ca^2+^]_i_ change in cells incubated with 0.1 mM gentamicin sulfate for 10, 20, and 30 min. Gd 10 (n=25/5), Gd 20 (n=25/5), and Gd 30 (n=25/5) correspond to the [Ca^2+^]_i_ change in cells incubated with 0.3 mM gadolinium, without the addition of gentamicin. EGTA 10 (n=14/7), EGTA 20 (n=6/3), and EGTA 30 (n=6/3) indicate the [Ca^2+^]_i_ variations obtained in the presence of EGTA solution containing 0.1 mM gentamicin sulfate. *P<0.001 compared to C; ^&^P<0.02 compared to G 10; ^#^P<0.002 compared to G 20; ˆP<0.002 compared to G 30 (ANOVA). AUF (arbitrary units of fluorescence).

Incubation of MDCK-C11 cells monolayers with 0.1 mM gentamicin sulfate determined a significant increase of [Ca^2+^]_i_ over time as observed in the representative recording shown in Figure 1. When MDCK-C11 cells monolayers were incubated for 10 min, intracellular calcium increased from 100 to 117.3±23.7% (P<0.05). This increase reached 130.3±27.3% after 20 min and was sustained after 30 min, where the difference from baseline recording was 138.1±32.9% (Figure 2). When the cells were incubated with 0.3 mM Gd^3+^, a CaSR agonist, the cytosolic ionized calcium increased to 107.9±19.6% after 10 min, 111.9±9.3% after 20 min, and 116.1±8.0% after 30 min. These values were significantly lower than those observed with gentamicin, as shown in Figure 2. The mechanisms for the increase of [Ca^2+^]_i_ observed might be an influx of extracellular Ca^2+^ or activation of intracellular signaling pathways that promote release of calcium from the endoplasmic reticulum. In the absence of extracellular calcium (from the addition of 1.8 mM EGTA to medium containing gentamicin), the [Ca^2+^]_i_ was significantly lower than control values at all exposure times (Figure 2). After incubation of MDCK-C11 cells for 10 min with the gentamicin solution containing 50 mM BAPTA, an intracellular calcium chelator, the [Ca^2+^]_i_ decreased to near baseline values (96.18±1.03%).

### Cell viability and cellular cytotoxicity

**Figure 3.**
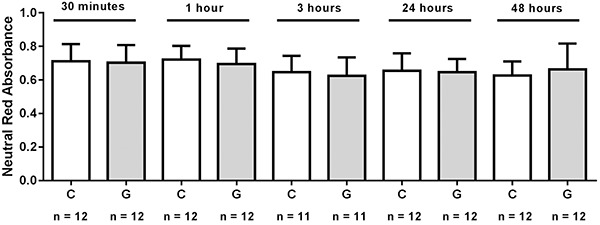
Neutral red absorbance by MDCK-C11 cells exposed to 0.1 mM gentamicin sulfate (G) compared to control (C) (P>0.05, Student’s *t*-test for paired samples).

MDCK-C11 cells viability was determined by the neutral red method, which evaluates the cell endocytic ability by measuring the absorbance of the dye. In these experiments, we evaluated changes in endocytosis capacity of the cells by exposing them to 0.1 mM gentamicin at different times: 30 min, 1, 3, 24, and 48 h (Figure 3). No significant difference was found between treated and control groups.

**Figure 4.**
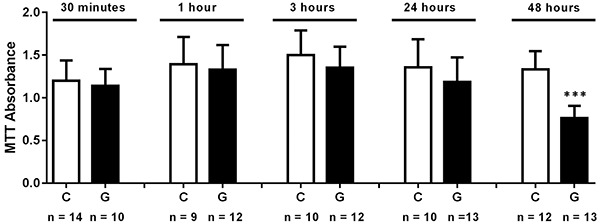
MTT absorbance by MDCK-C11 cells exposed to 0.1 mM gentamicin sulfate. ***P<0.0001 compared to control (C) (Student’s *t*-test for paired samples).

Using the MTT method, it was determined if gentamicin addition to MDCK-C11 cells may cause some toxic effect. From the measurement of the formazan crystals absorbance, cell viability associated with cytotoxicity in MDCK C11 cells was evaluated (Figure 4). When the cells were maintained for 48 h with gentamicin, the absorbance of the formazan crystals was significantly lower (0.76±0.14, n=13) than that observed in cells maintained in control situation (1.33±0.21, n=12), which indicated a reduction in their metabolic activity. A tendency of reduction in metabolic activity was also observed with 24 h of gentamicin exposure, but without statistical significance.

### Scanning electron microscopy

**Figure 5.**
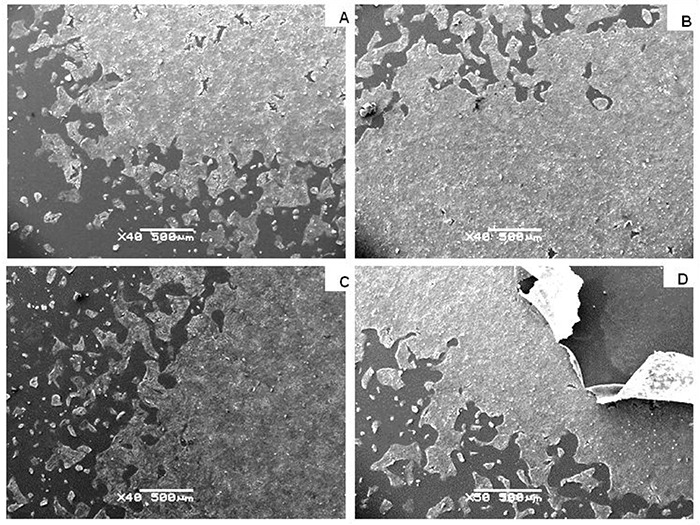
Ultrastructural analysis of MDCK-C11 cells by scanning electron microscopy. *A*: cells exposed to control solution for 3 h and *B*: for 24 h. *C*: cells exposed to 0.1 mM gentamicin solution for 3 h and *D*: for 24 h. Magnification bars: 500 μm.

**Figure 6.**
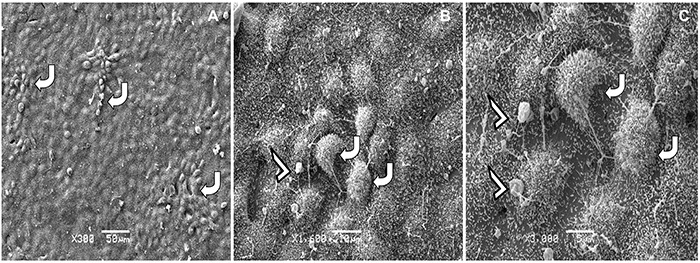
Ultrastructural analysis of MDCK-C11 cells by scanning electron microscopy. Panels *A*, *B*, and *C*: cells exposed to 0.1 mM gentamicin solution for 1 h. Magnification: *A*, ×300, 50 μm; *B*, ×1600, 10 μm; *C*: ×3000, 5 μm. Curved arrows: cell colonies that have a more protruding aspect. Arrow head: fibrillar material.

The ultrastructural analysis of the cells by scanning electron microscopy demonstrated that monolayers exposed to control solution (3 and 24 h) and those treated with gentamicin for 3 h showed that cells adhered well to substrate (Figure 5A, B, and C). Only when the cells were treated with gentamicin for 24 h were they loosened in a monolayer substrate (Figure 5D). After treatment, these cells did not lose intercellular adhesion, but lost adhesion to substrate. Figure 6 shows an epithelium structure change just after 1 h of gentamicin treatment (Figure 6A, B, and C). Some colonies have cells with a more protruding aspect. Furthermore, over the entire cell culture, the presence of fibrillar material deposited can be observed, which does not appear in longer periods of gentamicin exposure.

## Discussion

Like gadolinium, neomycin, and tobramycin, gentamicin is also known as an agonist of CaSR (7,19). Stimulation of CaSR activates phospholipase C, resulting in the production of diacylglycerol and inositol 1,4,5-trisphosphate, the latter of which releases Ca^2+^ from the endoplasmic reticulum. In this study, MDCK C11 cells incubated for 10 min with 0.1 mM gentamicin sulfate showed an increase in the intracellular Ca^2+^ level ([Ca^2+^]_i_) that was higher than that obtained with gadolinium. This finding indicated that gentamicin would have a more pronounced effect on the activation of CaSR. Moreover, in the presence of BAPTA, there was no change in the [Ca^2+^]_i_ level, even in the presence of gentamicin, because this drug is able to chelate all cytoplasmic Ca^2+^. An increase in the [Ca^2+^]_i_ level greater than 30% may be responsible for triggering the toxic effect of this aminoglycoside on MDCK C11 cells. This effect does not depend on the endocytic pathway, but occurs by stimulation of Ca^2+^ per Ca^2+^, because in the absence of extracellular Ca^2+^ (EGTA), the [Ca^2+^]_i_ level remained at the basal level.

In this study, we also evaluated the viability of MDCK-C11 cells exposed to gentamicin. Cells exposed to the antibiotic gentamicin showed no change in the uptake of neutral red dye, indicating that the endocytosis function was not impaired by the antibiotic. With regard to the evaluation of the cytotoxicity caused by gentamicin, MDCK-C11 cells showed a significant decrease in metabolic activity with 48-h exposure, which indicates metabolic stress.

As shown in Figure 2, gentamicin promoted an increase in the [Ca^2+^]_i_ level. A high concentration of Ca^2+^ released from the endoplasmic reticulum promotes a consequent high Ca^2+^ uptake by mitochondria. In these organelles, high Ca^2+^ levels activate apoptosis pathways (8). Therefore, the reduction in the metabolic activity of MDCK-C11 cells observed after 48 h of exposure can be interpreted as the beginning of an apoptotic process promoted by gentamicin. Further studies are necessary to confirm a hypothetical apoptosis induced by gentamicin in MDCK-C11 cells.

Scanning electron microscopy analysis showed that gentamicin promoted morphological and ultrastructural changes in MDCK-C11 cells. With longer exposure to aminoglycoside (24 h), cells failed to remain adhered, losing their adhesion to the extracellular matrix (ECM), possibly by modulation of the receptors that enable cell adhesion. Among several molecules related to ECM, the transmembrane glycoproteins integrins can be emphasized, which have α and β domains and are membrane receptors. These biomolecules interconnect the internal cell cytoskeleton with the ECM (20). Among the ECM proteins with which integrins intersect are fibronectin, laminin, and vitronectin. This interaction is modulated via cytoplasmic proteins such as vinculin, talin, and α-actinin, which interact with integrins and cytoskeletal proteins, and are involved in dynamic adhesion, invasion, migration, and proliferation (21). Syndecan is a cell surface proteoglycan, which can act as a co-receptor for growth factors and ECM proteins by increasing the affinity of these molecules to their specific receptors; it also participates in cell adhesion on focal contacts with integrins and focal adhesion kinase, connecting the ECM to the cytoskeleton. Thus, it is believed that the loss of adhesion to the substrate in MDCK-C11 cells treated for 24 h with gentamicin would be related to any changes in the activity of membrane proteins such as integrins and syndecan.

The dose of gentamicin used in the present study (0.1 mM gentamicin sulfate) was smaller than that used in other *in vitro* studies. However, this dose of gentamicin was shown to alter metabolism and morphology of MDCK-C11 cells. Gibbons et al. (22) showed that gentamicin induces cell death in OK cells (proximal tubular cells) at a low concentration (50 µM). In addition, some studies have shown that gentamicin is able to cross the plasma membrane (via cation channels) of renal tubular cells, including MDCK. Thus, induction of cytotoxicity caused by gentamicin may possibly occur at very low doses of the antibiotic (23).

In conclusion, our results demonstrated that gentamicin was capable of promoting a significant increase in [Ca^2+^]_i_ levels in MDCK C11 cells, which is dependent on the presence of Ca^2+^ in the extracellular medium. The gentamicin-induced increase in [Ca^2+^]_i_ levels was significantly higher compared to the increase caused by a CaSR agonist, gadolinium. Gentamicin altered metabolic activity and morphology of cells that resemble those of intercalated cells of the distal nephron. Intercalated cells play an important role in the acid-base balance of the organism, contributing to pH homeostasis by reabsorbing bicarbonate and secreting the hydrogen ion in accordance with the body's needs. The findings of the present study demonstrate, for the first time, that the toxic effect of gentamicin on intercalated cells can lead to changes in the metabolism of these cells. These changes promote cell death, which could contribute to the progression of renal failure.
